# Prestimulus amplitudes modulate P1 latencies and evoked traveling alpha waves

**DOI:** 10.3389/fnhum.2015.00302

**Published:** 2015-05-27

**Authors:** Nicole A. Himmelstoss, Christina P. Brötzner, Andrea Zauner, Hubert H. Kerschbaum, Walter Gruber, Julia Lechinger, Wolfgang Klimesch

**Affiliations:** ^1^Department of Psychology, University of SalzburgSalzburg, Austria; ^2^Center for Cognitive Neuroscience, University of SalzburgSalzburg, Austria; ^3^Department of Cell Biology, University of SalzburgSalzburg, Austria

**Keywords:** traveling waves, P1 latencies and topography, alpha waves, background excitation, frequency change

## Abstract

Traveling waves have been well documented in the ongoing, and more recently also in the evoked EEG. In the present study we investigate what kind of physiological process might be responsible for inducing an evoked traveling wave. We used a semantic judgment task which already proved useful to study evoked traveling alpha waves that coincide with the appearance of the P1 component. We found that the P1 latency of the leading electrode is significantly correlated with prestimulus amplitude size and that this event is associated with a transient change in alpha frequency. We assume that cortical background excitability, as reflected by an increase in prestimulus amplitude, is responsible for the observed change in alpha frequency and the initiation of an evoked traveling trajectory.

## Introduction

Brain oscillations as measured by the EEG are manifested by rhythmic fluctuations in amplitude size that show a relation to membrane currents of masses of neurons (for a review, cf. Buzsáki et al., 2012). They reflect phases of low and high excitability which are associated with phases of decreased and increased firing rate. This basic timing mechanism of oscillations has been well described for a variety of different rhythms (Buzsáki, 2006; Steriade, 2006). The general hypothesis is that temporal aspects of neuronal firing, which are crucial for information transmission, are largely and causally organized by oscillations (Buzsáki and Draguhn, 2004; Romei et al., 2010). Because cortical neurons form large networks (Hagmann et al., 2008), information transfer between neurons implies a spatiotemporal distribution of oscillations which is manifested by traveling waves (Wu et al., 2008).

Traveling waves have been observed very early in electrophysiological research (Adrian and Matthews, 1934; Petsche and Marko, 1955; Hughes, 1995) and have been documented on all levels of measurements, including the synaptic level (Williams, 1992; Wu et al., 2008; Nadasdy, 2009), the local field potential recorded from microelectrodes in animals and humans (Rubino et al., 2006; Takahashi et al., 2011; Nauhaus et al., 2012), the ECoG, EEG, and MEG (Freeman and Barrie, 2000; Alexander et al., 2013) in a large variety of studies focussing, e.g., on sensory encoding processes (Ermentrout and Kleinfeld, 2001; Dong and Olson, 2008), information transfer between cortical regions (Rubino et al., 2006) and methodological aspects (Alexander et al., 2006; Hallatschek, 2010). They have been observed in the ongoing EEG (Bahramisharif et al., 2013), in evoked potentials (Alexander et al., 2006, 2013; Klimesch et al., 2007a), in the flicker induced steady state evoked potential (Silberstein et al., 1990; Burkitt et al., 2000), and in the sleep EEG (Massimini et al., 2004). The physiological process underlying traveling waves represents a crucial feature for theories on encoding (Nadasdy, 2010) and for brain theories describing the functional interplay between different neuronal networks (Nunez, 2000; Nunez and Srinivasan, 2014). The most general finding is that traveling waves are ubiquitous in the ongoing EEG, but occur intermittently in episodes (Patten et al., 2012). A special and important phenomenon are evoked traveling waves (Alexander et al., 2006, 2009; Klimesch et al., 2007a) because they link the ongoing EEG with properties of the event-related potential (ERP). In a study, focusing on the within trial spatial phase distribution, Alexander et al. (2013) have demonstrated that the trial averages of traveling waves reproduced the topography of ERPs. Particularly interesting is the observation that topographical latency differences of the P1 component can be described in terms of an evoked traveling alpha wave with a posterior lateral to medial traveling movement in lexical and semantic decision tasks (Klimesch et al., 2007a; Fellinger et al., 2012; Zauner et al., 2014). This means that the phase topography of evoked alpha, but not that of other EEG frequencies, (Gruber et al., 2005; Klimesch et al., 2007a) coincides with the topography of the P1 components. Since the pioneering work of Berger (1929), alpha waves are known as an important phenomenon of the ongoing EEG. An interesting question, arising from these observations is which processes underlie the transition from an ongoing to an evoked traveling wave. This is the question, we ask in the present study.

A simple method for detecting an evoked traveling wave is based on two requirements. First, an evoked component must exhibit a spatiotemporal distribution that allows for determining a leading and trailing electrode site. Second, the evoked component must have a dominant frequency characteristic within a typical frequency range (such as e.g., alpha). The P1 component fulfills these requirements. Several studies have shown that the P1 has a typical spatiotemporal distribution and a characteristic frequency in the alpha frequency range (Gruber et al., 2005; Klimesch et al., 2007b; Fellinger et al., 2012). The leading site is that site with the shortest P1 latency, whereas the trailing site is that with the longest P1 latency. An evoked traveling wave has – by definition – a clear onset of traveling movement peri- or poststimulus.

**FIGURE 1:**
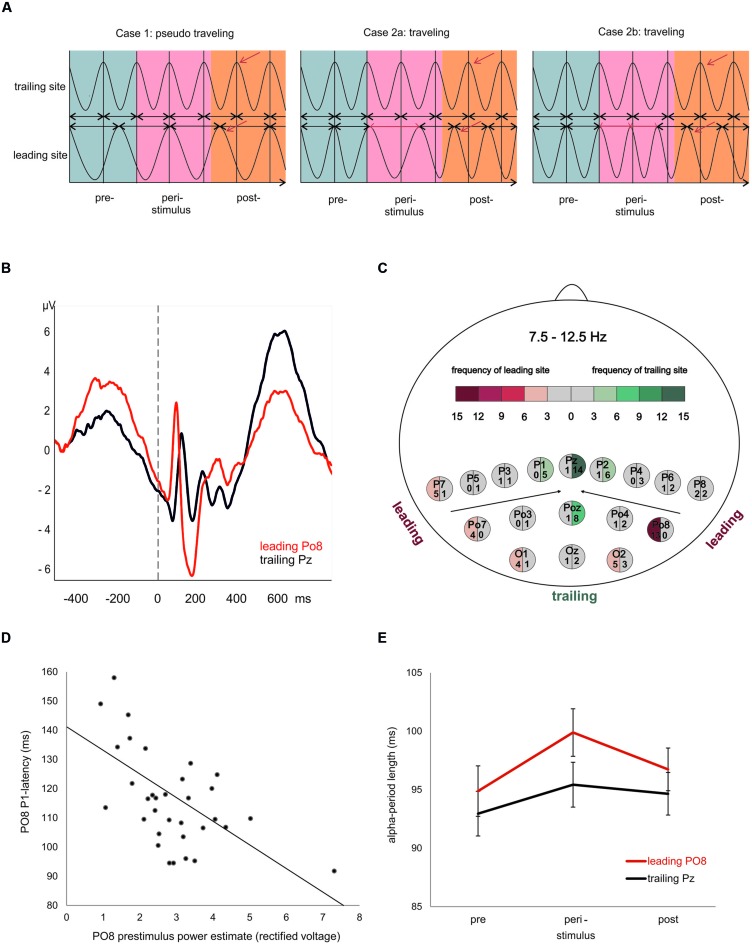
**Evoked traveling alpha waves: theoretical considerations and empirical findings. (A)** The crucial factor for the appearance of a traveling wave is frequency. If at different sites alpha oscillates with slightly different frequencies (left), a topographic phase gradient – indicating a traveling wave – will appear (left). In such a case, however, if the peri- and or poststimulus wave lacks any (stimulus and/or event related) modulation, the phase gradient will vary randomly (with respect to stimulus onset) and averaging over trials will not exhibit an evoked traveling wave. The next two examples (middle and left) reflect cases, where oscillatory frequency is identical at different sites during the prestimulus period, but is modulated during or after stimulus presentation. Either a transient increase (middle) or decrease (right) is capable of inducing a systematic traveling trajectory that appears as systematic evoked phase gradient after averaging over trials. **(B)** ERPs at the most frequent leading and trailing electrode. Note the large prestimulus wave and short P1 latency at the leading site. **(C)** Map of electrodes and the color coded frequencies, reflecting the number of cases each electrode was classified as leading or trailing. **(D)** Significant correlation (*r* = -0.56) between prestimulus power and P1 latency at the most frequent leading electrode Po8. **(E)** Average period durations of individual alpha frequency for the most frequent leading and trailing sites estimated for a pre-, peri-, and poststimulus interval. Note the significant increase in alpha period (reflecting a transient decrease in frequency) in the peristimulus period. This finding agrees with the situation described in the middle of **(A)** and suggests that a transient slowing in alpha is responsible for the poststimulus traveling trajectory.

What kind of physiological process would be capable of inducing a topographical specific frequency change at the leading site? To our knowledge, this question has not yet been addressed in an empirical study. In an attempt to answer this question, we proceed from the idea that a traveling trajectory can be induced by a transient frequency change. When we have exactly the same oscillatory frequency at two recording sites, the spatiotemporal phase relationship will remain constant. If, however, the oscillatory frequency exhibits a transient change at one of the two sites, a clear traveling trajectory may emerge as illustrated in **Figure 1A**. This transient change in frequency, which is manifested by a change in the period of one or more oscillatory cycles, can be understood as a special type of phase ‘reset’ (cf. Cases 2a,b in **Figure 1A**). We assume that this frequency change occurs at the leading site, because it is that site from where the traveling wave spreads to other sites.

What could be the physiological mechanism underlying a transient frequency change? We proceed from the idea that a change in ‘background’ excitation and/or inhibition might be related to a change in oscillatory frequency (Nunez and Srinivasan, 2014). For slow EEG fluctuations (with a frequency of about 1 Hz and slower) there is good evidence that they reflect cyclical variations in the excitability of neuronal ensembles (e.g., Bishop, 1933; Steriade et al., 1993; Contreras et al., 1996; Sanchez-Vives and McCormick, 2000). This research has shown that action potentials are generated during an excitatory up state but not during an inhibitory down state (Amzica and Steriade, 1997). The scalp surface polarity of such an up and down state is difficult to predict, because it depends (beside other factors) on the exact location of the source. Nonetheless, based on these findings one may speculate, that the magnitude of a slow deflection (in the sub-delta or delta frequency range) reflects a change in background excitation and is correlated with P1 latency in a sense, that a large deflection is related to a large frequency change and a large change in P1 latency. There is yet another, additional, possibility that must be considered. Research on rat hippocampus gamma oscillations has shown that instantaneous oscillatory frequency (as measured in terms of the duration of the gamma period in single trials) depends on the extent of excitation and inhibition (Whittington et al., 1995; Traub et al., 1996; Atallah and Scanziani, 2009). In an interesting study by Atallah and Scanziani (2009) clear evidence was found that fluctuations in (instantaneous) gamma amplitude reflect changes in synaptic excitation and are associated with fluctuations in (instantaneous) gamma period. The basic finding was that an increase in amplitude is closely associated with a lengthening in the immediately following period, and – vice versa – a decrease in amplitude is associated with a shortening in the immediately following period. We refer to this finding as ‘cycle to cycle fluctuations in amplitude and period.’ It is manifested by a significant positive correlation between amplitude and period on a cycle per cycle basis.

These two mechanisms, slow waves (associated with up and down states) and cycle to cycle fluctuations in oscillatory amplitude size and period length must not be considered mutually exclusive interpretations. It might well be possible that slow wave components are associated with increased oscillatory cycle to cycle fluctuations in different frequency ranges.

The measurement of cycle to cycle fluctuations of a frequency of interest – in our case the alpha band - requires a specific analyzing procedure. First, for single trials, the time points of peaks and troughs are identified for the band pass filtered data. Then, and most importantly, these time points are used to determine the amplitudes of peaks and troughs in the raw data. The reasons for this procedure is, that alpha band pass filtering abolishes the influence of (and a possible interaction with) slow components and in addition tends to reduce asymmetric and short lasting amplitude fluctuations. This method, which determines the amplitudes of peaks and troughs in the raw data, was suggested by researchers who found evidence for asymmetric alpha amplitude fluctuations (e.g., Nikulin et al., 2007; Mazaheri and Jensen, 2008) will be applied here in addition to traditional ERP analyses.

In the present study, we used a semantic (living vs. non-living) judgment task that already proved useful to investigate evoked alpha waves (Zauner et al., 2014). One of the basic findings was that words with many semantic features tend to reduce the latency difference between leading and trailing sites but for yes response trials (to living objects) only. Here, we want to test the above described hypotheses regarding the predicted association between the amplitude of a slow prestimulus ERP deflection on the one hand and a change in P1 latency at the leading site on the other hand. In addition, we want to test, whether a transient frequency change in alpha can be observed at the subject level (i.e., for the averaged EEG data as reflected by the ERP) but also on the single trial level (i.e., for the not averaged data). This latter method aims to detect the single trial P1 and to analyze alpha amplitude to period fluctuations for several cycles immediately preceding the P1.

## Materials and Methods

### Subjects

A sample of 34 subjects (all different from those, used in Zauner et al., 2014), students of the University of Salzburg, participated in the present study after giving informed consent. The sample consisted of 14 male (mean age = 25.1 ± 2.5 years) and 20 female subjects (mean age = 23.2 ± 3.4 years). These subjects had normal or corrected-to-normal vision, did not report neurological diseases and were not on psychotropic medication. All subjects were compensated by course credits. The experiment was conducted according to the code of ethics (World Medical Association, 1996) and was approved by the Ethics Committee of the University of Salzburg.

### Stimulus Material and Task

The stimulus material and task was identical with a previously published study by Zauner et al. (2014). Here we give just a brief outline of the most important features of the stimulus material and task design. Subjects performed a semantic (living vs. non-living) judgment task. A total of 280 German words were presented in a randomized sequence. For each word, subjects had to decide, whether it represents a living or non-living object. The living and non-living category consisted of 140 words each, which were subdivided into 70 words with a large number of visual-semantic features (NOF+) and 70 words with a small NOF-. An example for a NOF+ word is eagle, an example for a NOF- word is hamster. The words were taken from McRae et al.’s (2005) “semantic feature production norms” and were translated into German.

Subjects were seated in a comfortable chair in front of a computer monitor (75 Hz refresh rate) at a distance of about 130 cm. The fixation cross was replaced by a word written in upper-case-letters either belonging to the living or non-living category. The word was presented for 1000 ms in dark gray (horizontal angle: 2,8∘–4,3∘; vertical angle = 0,66∘) within a bright gray box (9,7∘ × 2,6∘) to ensure comfortable reading and to hold visual surface features constant between trials. The interval between the onset of the fixation cross and the onset of the word varied between 400 and 600 ms in 50 ms intervals in order to reduce onset expectations. The intertrial interval varied between 1900 and 2100 ms. Subjects indicated by button press on the keyboard with their left index finger when the word denoted a living (yes-response) and the right index finger when the word denoted a non-living object (no-response).

### EEG Recordings

We used a 64-channel BrainAmp amplifier (BrainProducts, Inc., Gilching, Germany) for EEG recording. Signals were online referenced against the nose and subsequently (off-line) re-referenced to digitally averaged [(A1 + A2)/2] ear lobes. Recording bandwidth was set from 0.016 to 100 Hz and a notch filter at 50 Hz. Signals were digitized at a sampling rate of 1000 Hz 60 Ag–AgCl-electrodes were mounted using an EasyCap. For the present study we used only 17 posterior electrodes (P7, P5, P3, P1, Pz, P2, P4, P6, P8, PO7, PO3, POz, PO4, PO8, O1, Oz, O2) for data analysis. Impedances were kept below 8 kΩ. To control for vertical and horizontal eye movements two bipolar EOG-channels were mounted. After re-referencing, epochs containing eye artifacts were corrected by the Gratton et al. (1983) and muscle artifacts were rejected. BrainVisionAnalyzer (BrainProducts, Inc.) was used for data analyses. Epochs consisted of EEG segments ranging from -600 to 1000 ms relative to the stimulus.

### Data Analysis: ERPs, Prestimulus Power, and Transient Changes in Evoked Alpha Frequency

For the calculation of ERPs we used two different filters, a broad band filter between 0.5 and 70 Hz. For alpha bandpass filtering we used an equiripple filter (7.5–12 Hz passband, 24 db at cutoff frequencies of 6 and 14 Hz). Prestimulus power was determined as the average of rectified voltage within a time window of – 100 to 200 ms relative to stimulus onset (0 ms) for the broadly filtered data (i.e., the raw data), a subdelta band (0.5–2.5 Hz), and the alpha band.

The alpha filtered data were used to calculate P1 peak latencies and amplitude. This was done because the P1 peak is easier to detect in the filtered data. In the ERPs of the broadly filtered data, evoked beta activity can be observed that leads to double peaks in the P1 time window and makes the detection of the P1 ambiguous. For the calculation of evoked frequency changes we used a narrow alpha band pass filter (with a width of 2 Hz) that was adjusted to individual alpha peak frequency (IAF) as center frequency (IAF ± 1 Hz). IAF was determined as the spectral component within an extended alpha frequency range of 7–14 Hz which showed the largest power during a pre-task eyes closed resting period of 5 min. The obtained IAF’s were averaged over four electrodes (P3, P4, Po3, and Po4). The segmented data were averaged and peaks were detected semi-automatically.

The typical latency of the visual P1 is between 80 and 120 ms post-stimulus (cf. **Figure 1B** for an illustration of the P1 latency differences). The semi-automatic algorithm detects positive peaks within a defined time window. We used a window of 70–185 ms because we expected a large variation in P1 latency due to a traveling movement. The algorithm defines any positive going deflection as positive peak even if the absolute amplitude has a negative voltage. By visual inspection we accepted only those peaks which had a positive amplitude larger than 0.5 μV. This procedure was done separately for each of the four conditions (living NOF+, NOF- and non-living NOF+, NOF-). In cases of missing values we used the respective values of the remaining condition(s). For each subject, response type (yes and no response to words denoting a living or non-living object) and semantic feature condition (NOF+ and NOF-), we determined that electrode with the shortest P1 latency and that with the longest P1 latency. These electrodes are referred to as leading and trailing sites in the following. As depicted in **Figure 1C**, PO8 was the most frequent leading site and Pz the most frequent trailing site. We then calculated the latency differences between the leading and trailing sites as a gross measure of the traveling movement of the evoked alpha wave for each subject, response type and feature condition.

We also used a peak detection procedure to determine transient changes in alpha period (as measure of ‘instantaneous’ alpha frequency) around stimulus onset. For this purpose we used the averaged alpha filtered data for the leading electrode and determined the interpeak latencies between six consecutive positive peaks. As starting point, we used the second positive alpha peak that occurred poststimulus. From this peak we calculated the interpeak latencies between six consecutive positive peaks (i.e., comprising the second positive peak poststimulus plus five consecutive peaks) going backward in time into the prestimulus interval. This procedure yielded five different period estimates for each subject, averaged over all conditions. In the following, the first period value is referred to as poststimulus period. The next following value was termed peristimulus period. The last three values were averaged and termed prestimulus period. This was done to reduce differential influences of the filter stemming from evoked components.

### Single Trial Data Analysis: Prestimulus Amplitude and Transient Changes in Alpha Frequency

**FIGURE 2:**
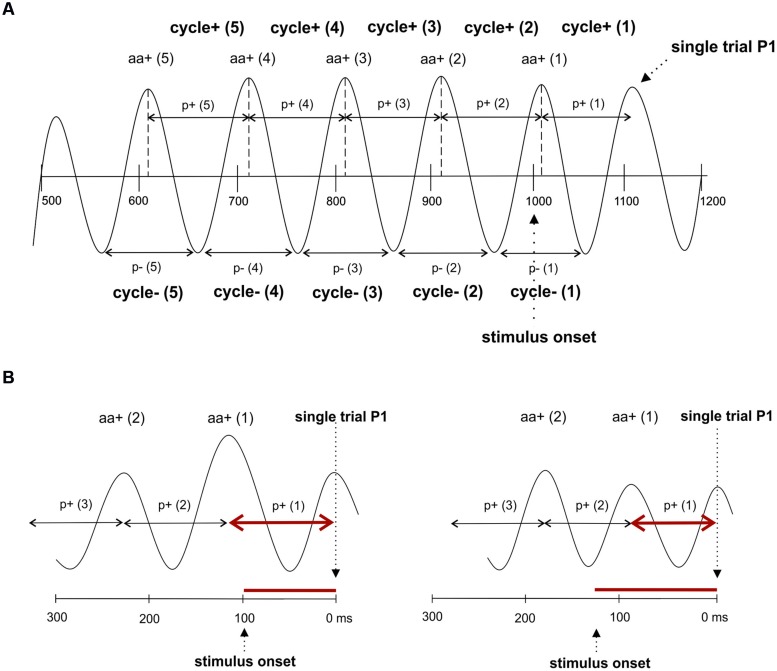
**The single trial analysis focuses on consecutive cycles preceding the P1. (A)** The interpeak latencies between positive alpha peaks and negative alpha peaks were measured. Note that this procedure yields different values for cycles determined for positive and negative peaks. The notation of the different variables that were measured are depicted. **(B)** Due to the fact that interpeak latencies (i.e., the length of a period of an alpha cycle) are measured relative to the single trial P1, but P1 latencies relative to stimulus onset, a correlation between amplitude size and the two latency measures go into different directions: An increase in oscillatory period in cycle 1 is associated with a shortening of the P1 latency (left), whereas a decrease in the period is associated with a lengthening of the P1 latency (right).

To test, whether fluctuations in prestimulus amplitude in single trials are associated with fluctuations in P1 latency, we used a recently suggested method for the detection of single trial P1 components (Gruber et al., 2014). This method is based on the hypothesis that ongoing alpha develops more or less seamlessly (but with some jitter) into the P1 component. It selects those trials, where a positive alpha peak can be observed within the time window of the P1 component of the ERP. The selection algorithm comprises four steps. First, we define a selection window as an interval of ±32 ms around the individual ERP P1 latency at the leading electrode PO8. This window – referred to as window 1 in the following – was chosen to be long enough to cover a half cycle of slow alpha of about 8 Hz. Second, within this window, we search for a positive voltage alpha peak (the single trial P1) in the alpha filtered data. The search criterion is a positive peak with positive amplitude in the individually determined 64 ms time window. Third, those trials for which the peak detection yielded a positive result constitute the subset of selected trials. It comprises those trials, in which ongoing alpha develops more or less seamlessly (within the defined selection window) into the P1. For these trials – which are simply termed *selected trials* in the following – the single trial P1 latencies (the latency of the selected positive alpha peak) were determined. The average number of selected trials per subject was 172 out of 206. This means that 83% of all trials were alpha phase aligned to the individual P1 component. This subset of trials constitutes the selected trials. The remaining 17% of trials are those that are not phase aligned to the P1. They are termed *rejected trials*. For the investigation of fluctuations in alpha frequency in single trials, we used the selected trials, obtained in step three as described above. In these data, we used the single trial P1 as starting point for determining the interpeak latencies between five consecutive positive peaks going backward in time into the prestimulus interval as illustrated in **Figure 2A**. This procedure allowed us to measure the length of five consecutive alpha periods by going backward in time from the single trial P1 to the prestimulus period. These are termed p+(1) … p+(5) for the periods between positive alpha peaks and p-(1) … p-(5) for the respective periods between negative alpha peaks starting from the first negative amplitude preceding the single trial P1 (cf. **Figure 2A**).

To study fluctuations in instantaneous EEG, amplitudes that are phase locked to the positive alpha peaks of each of the five cycles (preceding the single trial P1), we determined for each trial the time points of these peaks. At each of these five time points we determined the amplitude of the raw EEG data and the alpha filtered data. For the raw (r) data, the five amplitude estimates are termed ar+(1) … ar+(5), denoting the first … fifth raw amplitude (coinciding with the positive alpha peak) preceding the single trial P1. For the alpha filtered data (a), the five amplitude estimates are termed aa+(1) … aa+(5), denoting the first … fifth positive alpha amplitude preceding the single trial P1. The same procedure was applied for the amplitudes at the negative alpha peaks and the respective values are termed ar-(1) … ar-(5) and aa-(1) … aa-(5), respectively.

#### Single Trial Data Analysis: Control Analysis

The above described procedure aims at identifying those trials that exhibit a more or less seamless development of alpha phase with respect to the P1 component of the ERP. As a consequence, trials not meeting this criterion (which depends on the width of the selection window) are rejected. In order to test, whether the expected cycle to cycle fluctuations in amplitude and period depend on the width of the selection window, we ran a control analysis with an extended selection window (of ±50 ms), which was chosen to be long enough to cover more than a half cycle of slow alpha of about 8 Hz. This selection window of the control analysis is referred to as window 2 in the following. Because with this broad window more than one positive peak can appear, we selected that positive peak as single trial P1 which is closest to the individual P1 of the ERP. In contrast to the selection procedure with time window 1, which yielded 17% rejected trials, the percentage of rejected trials with time window 2 was only 2%.

### Statistical Analysis

We first wanted to determine, whether the present study replicates the findings from Zauner et al. (2014). For this purpose, we used a two way ANOVA with the factors response type (yes and no responses) and semantic condition (NOF+/-). The dependent measure was latency difference between leading and trailing sites. Across subjects, correlations were calculated between prestimulus power and mean P1 latency (averaged over all conditions) for the most frequent leading electrode PO8. To test for transient changes in instantaneous alpha frequency, we calculated *a priori t*-tests to test the hypothesis that the predicted transient change in alpha period occurs selectively for the peristimulus period. In addition, we performed a two way repeated measures ANOVA with electrode (Pz, PO8) and time (pre-, peri-, and poststimulus period) as factors. The three levels of factor time represent (i) the first poststimulus alpha period (measured from the second positive evoked peak that appears after stimulus onset to the next positive peak, by going backward in time), (ii) the next period (backward in time) constitutes the peristimulus period. (iii) The prestimulus period is the average of the three consecutive periods that follow the peristimulus period (again determined by going backward in time). The dependent measure was the duration of the alpha period determined for IAF.

For the single trial analysis, we calculated correlations between the single trial P1 latencies, the five preceding amplitude estimates at the positive and negative alpha peaks, and between the amplitude estimates and the immediately preceding periods. Because our hypothesis predicts a positive association between amplitude size and period, we used one-sided significance values at the 5% level. We also calculated one way ANOVA’s with factor TIME (with five levels, denoting the first … fifth cycle preceding the P1) and amplitude/period as dependent measures.

## Results

### Behavioral Data and Replication Analysis

The overall mean RT was 688.94 ms and the mean percentage of correct responses was 96%. The RT’s in the four conditions, yes responses NOF+/-, and no responses NOF+/-, were in that order: 648.9 (SD = 58.4), 677.5 (SD = 63.9), 704.3 (SD = 79.0), 725.1 (SD = 86.4). IAF varied between 8.1 and 12.9 Hz around a mean of 10.3 Hz (SD = 1.01).

The two way ANOVA with semantic condition (NOF+/-) and response type (yes vs. no response) as factors and latency difference (between leading and trailing sites) as dependent measure yielded a significant interaction [*F*_(1,33)_ = 4.987, *p* < 0.05). The main effects did not reach significance. Inspection of the respective means indicates that a large number of features (NOF+) tends to increase latency differences for no responses, but tends to decrease latency difference for yes responses. These findings replicate the basic aspects of the Zauner et al. (2014) study.

### Prestimulus Power, P1 Latency, and Peristimulus Changes in Alpha Period

Visual inspection of the ERPs at the most frequent leading and trailing sites reveals large differences in prestimulus power (for the raw data) and P1 latency as depicted in **Figure 1B**. The average P1 latencies are 116.0 ms (SD = 16.3 ms) for PO8 and 135.8 ms (SD = 10.4 ms) for Pz. The calculation of correlations between the respective variables showed a highly significant association for the leading electrode (PO8; *r* = -0.56, *p* < 0.01) but not for the trailing electrode (Pz; *r* = -0.12). As shown in **Figure 1D**, at PO8, large prestimulus power is significantly associated with short P1 latencies. The correlations between PO8 P1 latency and the subdelta and alpha filtered prestimulus power yielded a significant result for the subdelta power only (*r* = -0.53; testing for symmetrical distributions of both variables, using the Kolmogorov–Lillieforce procedure, yielded no significant deviation). This finding suggests that the critical component for the correlation is a slow wave in the subdelta frequency range. Furthermore, dependent *t*-tests revealed a larger P1-amplitude for Po8 electrode compared to Pz electrode [*t*_(33)_ = 3.198, *p* = 0.003).

Changes in alpha period are depicted in **Figure 1E**. *A priori t*-tests showed that only the peristimulus alpha period was longer for Po8 as compared to Pz [*t*_(33)_ = 2.274, *p* = 0.030]. The pre- and poststimulus alpha- period length did not differ significantly. The two-way repeated measures ANOVA yielded a significant main effects for electrode site [leading vs. trailing, *F*_(1,33)_ = 9.178, *p* = 0.005] and time [*F*_(2,66)_ = 3.864, *p* = 0.027]. The interaction, however, did not reach significance. The significant main effect suggests slightly different frequencies at the two electrodes. However, the calculation of a *t*-test between IAF at Pz and PO8 (as measured during the resting period) did not show significant differences.

### Single Trial Analysis of Peri-/Prestimulus Amplitude

The single trial analysis focused on two different, but closely interrelated questions. One question refers to the association between single trial P1 latency and the magnitude of peri-/prestimulus amplitude, another to single trial fluctuations in amplitude and period.

#### Peri-/Prestimulus Amplitude and Single Trial P1 Latency

The correlations between the single trial P1 latencies and the amplitude estimates of the raw EEG [ar+(1) … ar+(5); ar-(1) … ar-(5)] at the time points of the five preceding positive and negative alpha peaks are summarized in **Table 1A**. The results for positive peaks show that 22 of the 34 subjects exhibit significant negative correlations between latency and ar+(1). These correlations indicate that an increase in ar+ is associated with a decrease in the single trial P1 latency. The respective correlations with the amplitudes at the negative alpha peaks are somewhat weaker, but exhibit an analogous relationship. The larger the magnitude of the negative amplitude [ar-(1)], the shorter is P1 latency.

**Table 1A:** Correlations between single trial P1 latencies and peri-/prestimulus amplitudes.

	Positive alpha peaks					Negative alpha peaks				
	ar+(1)	ar+(2)	ar+(3)	ar+(4)	ar+(5)	ar-(1)	ar-(2)	ar-(3)	ar-(4)	ar-(5)
**Window 1**					
Negative	22	0	8	8	5	1	1	2	1	3
Positive	0	1	1	1	0	11	5	4	2	3
**Window 2**					
Negative	27	0	12	6	3	1	4	3	3	4
Positive	0	5	0	2	1	12	5	0	3	1

*Number of significant negative and positive correlations with single trial raw EEG amplitudes at positive alpha peaks [ar+(1) … ar+(5)] and negative alpha peaks [ar-(1) … ar-(5)]*.

#### Single Trial Fluctuations in Amplitude and Period

When we focus on the cycle per cycle correlations between amplitude estimates [(ra+(1) … ra+(5) and ra-(1) … ra-(5)] and the respective, corresponding single trial periods [p+(1) … p+(5) and p-(1) … p-(5)] we see primarily positive correlations for positive amplitudes and negative correlations for negative amplitudes. These findings provide evidence for single trial fluctuations in amplitude and period and show that an increase in the magnitude of the raw amplitude tends to increase the period for the immediately following cycle.

It must be noted that the positive association between amplitude and period as depicted in **Table 1B** does not contradict the negative association between amplitude and single trial P1 latency as shown in **Table 1A**. The reason lies in the different ways P1 latency and period are measured. Latency is measured in relation to stimulus onset, but period is measured from the single trial P1 to the preceding amplitudes. Thus, as illustrated in **Figure 2B**, a long period preceded by a large amplitude is associated with a comparatively short latency, whereas a short period preceded by a small amplitude is associated with a long P1 latency.

**Table 1B:** Cycle to cycle fluctuations in amplitude and period.

	Positive alpha peaks					Negative alpha peaks				
	Cycle 1	Cycle 2	Cycle 3	Cycle 4	Cycle 5	Cycle 1	Cycle 2	Cycle 3	Cycle 4	Cycle 5
**Window 1**					
Negative	1	0	0	0	0	7	4	1	5	8
Positive	10	13	11	13	7	2	1	2	1	0
**Window 2**					
Negative	1	0	0	0	0	10	8	1	7	12
Positive	15	13	14	15	10	0	1	0	1	0

*Number of significant negative and positive correlations between raw EEG amplitudes at positive alpha peaks [ra+(1) … ra+(5)] and negative alpha peaks [ra-(1) … ra-(5)] and single trial periods measured between positive alpha peaks [p+(1) … p+(5)] and negative alpha peaks [p-(1) … p-(5)] for the first, second … fifth cycle preceding the P1*.

The correlations for the alpha filtered single trial amplitudes exhibit a very similar pattern of results. However, the number of significant correlations is much smaller than for the raw data.

#### Single Trial Analysis: ANOVA Results

**FIGURE 3:**
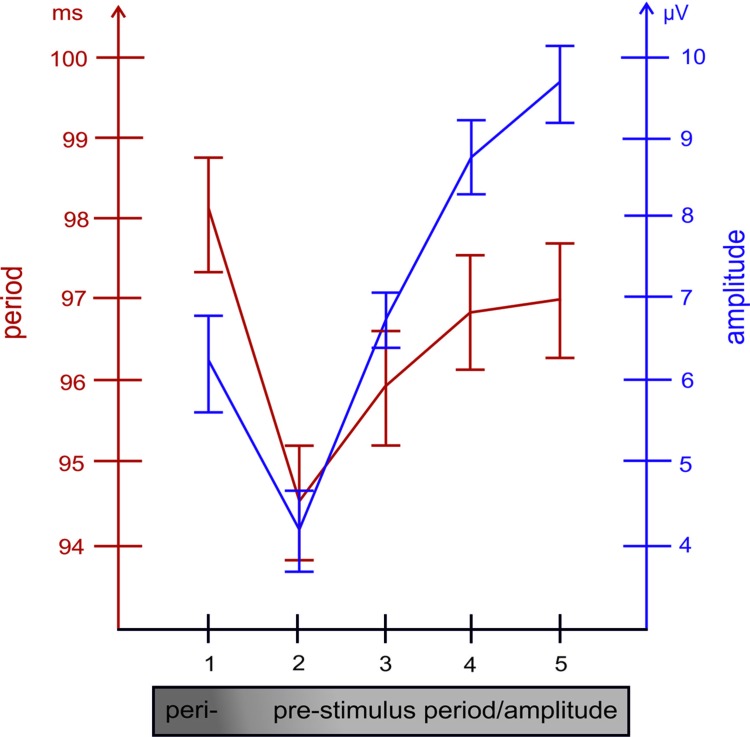
**The relationship between the length of the alpha period (measured between positive peaks) and the size of the amplitude (in the raw EEG, measured at the time point of positive alpha peaks) preceding the respective period, are depicted for each of the five cycles preceding the single trial P1**. Note the close association between both variables.

The results of the two ANOVA’s with TIME as factor and the subject averages for p+ and ar+ as dependent measures yielded significant effects [*F*_(2.10,69.44)_ = 12.63, *p* < 0.001 and *F*_(2.39,78.77)_ = 35.48, *p* < 0.001; the Greenhouse Geisser corrected df-values are depicted]. The findings show two different aspects. First, fluctuations in amplitude and period are highly significant between cycles. Secondly, the fluctuations in both variables, as depicted in **Figure 3**, co-vary in the same way, as was statistically documented by the correlational analysis as summarized in **Table 1B**. Amplitude size and the length of the immediately following period are perfectly associated: a large amplitude is followed by a long period and a small amplitude is followed by a short period. Most interestingly, the ANOVA with the subject averages for p- did not show significant differences. This suggests that the association between amplitude size and period is stronger for positive cycles.

#### Control Analysis

The control analysis showed very similar results, with a tendency to exhibit somewhat more significant correlations as a comparison of the findings for window 1 and window 2 in **Tables 1A,1B** reveals. Also, the respective ANOVA’s with TIME as factor and the subject averages for p+ and ar+ as dependent measures yielded very similar effects [*F*_(2.04,67.33)_ = 8.96, *p* < 0.001 and *F*_(2.29,75.67)_ = 33.84, *p* < 0.001; the Greenhouse Geisser corrected df-values are depicted]. Again, the ANOVA with the subject averages for p– did not show significant differences.

## Discussion

The most important findings are the significant correlations between prestimulus power and P1-latencies and the peristimulus increase in the alpha period at the leading electrode PO8 (cf. **Figures 1B,D,E**). In the ERP analysis, the latter effect is weak, because only *a priori t*-tests showed significant results. In the single trial analysis, however, highly significant differences in the magnitude of amplitudes and the length of alpha periods preceding the P1 were observed at the leading electrode as depicted in **Figure 3**. Most importantly, the fluctuations in alpha period are associated with amplitude (in the raw EEG, measured at the time point of alpha peaks) in a way that, a large amplitude is associated with a prolonged period in the immediately following cycle (cf. **Figure 2B** for a paradigmatic illustration of this finding). As an example, let us consider the first positive amplitude preceding the P1 and the first period (i.e., interpeak latency between the P1 and that first positive amplitude) as depicted in **Figure 3**. These two values are reflected by the first data points of the red and blue lines at time point 1 (i.e., at the time of the first positive alpha peak in the first cycle). As shown in **Figure 3**, amplitude size and period length co-vary over time (over cycles). It should be noted that the association between amplitude and alpha period is much weaker, if amplitudes are measured at the time points of the negative alpha peaks and interpeak latencies are calculated between the negative peaks (cf. **Figure 2A** for an illustration for the measurement of positive and negative cycles). We consider this an important finding, suggesting that amplitudes at positive and negative alpha peaks may be modulated differentially (cf. Nikulin et al., 2007; Mazaheri and Jensen, 2008) and that the modulation of the positive peaks is the critical factor that is associated with changes in P1 latency. We also have to emphasize that the results obtained for alpha amplitude size (instead of the amplitude of the raw EEG measured at the alpha peaks) are similar but much weaker as compared to the raw data. This latter finding most likely is due to the influence of the alpha band pass filter which abolishes slow drifts and tends to reduce the magnitude of short lasting, transient fluctuations in positive or negative peak amplitudes.

Taken together, these findings suggest that, in general, an increase in prestimulus amplitudes (of the raw EEG) is associated with a shortening of the P1 latency. The single trial analysis shows that amplitudes decrease from cycle 5 to cycle 2 (cf. **Figure 3**) in a similar way as power decreases (from about – 400 ms prestimulus) in the grand average ERP (cf. **Figure 3**). Because cycles (with a period around 100 ms) are counted relative to the P1, cycle 5 represents – in terms of time prestimulus – a time window around 400 ms. Thus, the time course of prestimulus amplitudes as measured at the positive alpha peaks in the single trial analysis and the time course of the slow component in the ERP match each other. In addition, the single trial analysis has revealed that an increase in the amplitude in cycle 1 (immediately preceding the P1) is associated with a lengthening in the alpha period (in that cycle) and a decrease in P1 latency. This latter finding resembles closely Case 2b (illustrated in **Figure 1A**) which describes a situation, where a peristimulus lengthening of the alpha period leads to a shortening in the P1 latency. Thus, as illustrated by Case 2a in **Figure 1**, a transient frequency change – elicited by a transient increase in the alpha period – may be the key factor for triggering a traveling wave with a well defined trajectory and that a topographical specific amplitude increase triggers that change.

It is also important to note that the results of the single trial analysis contradict the evoked model of ERP generation but also the phase reset model (at least in its most radical formulation). The reason is that 83% of all trials were found to be already aligned in phase in a way that voltage positive alpha peaks develop more or less seamlessly into the P1 (with a maximal jitter of ±32 ms). These findings replicate those of a visual target detection study (Gruber et al., 2014), which in addition has shown that phase aligned trials (i.e., selected trials) are associated with shorter detection times. We cannot rule out that the appearance of the fixation point, preceding the stimulus word, may play a role for the observed phase alignment. Because the interval was jittered (randomly between 400 and 600 ms) a direct influence of the fixation point seems unlikely. Seamless alpha is one argument against the evoked and phase reset model. An additional argument is that the rejected trials exhibit a single trial P1 that is counter phase to the positive peak in the P1 time window. This fact is in part due to the width of the selection window (±32 ms) which is close to a half period of slow alpha (with a frequency of about 8 Hz). If the P1 would be generated by fixed latency fixed polarity components, opposite polarities in single trials should not emerge within the time window of the P1. A reformulation of the phase reset model in the sense that phase is not abruptly reset but ‘reorganized’ in most trials (for a review cf. Klimesch et al., 2007c) would be very well in line with the results of the present study and those reported by Gruber et al. (2014).

Although the evoked model is not in line with the results of the single trial analysis of the leading site, the evoked model is capable of explaining latency differences between PO8 and Pz which we have interpreted in terms of a traveling wave. The theoretical basis for the evoked traveling wave model and that of the traditional evoked model are radically different. The former assumes that an event related phase reorganization at the leading site triggers an evoked traveling wave of different evoked components that move to the trailing site. In contrast, the traditional ERP model- based on the fixed polarity, fixed latency concept – assumes that different evoked components are generated at different sites within typical latency windows. As an example, according to the evoked traveling wave model, the C1 component (the negative peaks preceding the P1 at PO8 and Pz; cf. **Figure 1**) would be considered the negative peak of an evoked alpha wave that develops into the P1, and topographical latency differences are the result of the traveling movement. According to the evoked model topographical latency differences of one component may be due to a superposition with another component. With respect to our example it may be argued that the long latency of the P1 at Pz may be due to the influence of a pronounced C1 which delays the appearance of the P1. Because the C1 is usually larger at midline as compared to lateral sites (e.g., Clark et al., 1995), the delaying influence of the C1 on the P1 would be larger at midline as compared to lateral topographies. On the basis of our findings, we cannot rule out this alternative interpretation of the observed P1 latency differences between PO8 and Pz, although in an earlier study, we could show that the traveling movement also comprises the negative polarity peak preceding the P1 (Klimesch et al., 2007a). An empirical evaluation of these two conflicting interpretations would be possible in an experiment that aims to vary the C1 component according to the cruciform model which considers the different polarities stemming from activations of the lower versus upper banks of the calcarine fissure (e.g., Clark et al., 1995; Di Russo et al., 2002). For lower field presentations with a positive polarity C1, the evoked traveling model would predict a negative component within a half cycle of alpha. The evoked model would predict a superposition with a positive component within the typical latency window of the P1. A single trial analysis of the C1 component – in an analogous way as we have performed for the P1 – could clarify this question if lower and upper field presentations are compared.

Our findings are in perfect agreement with predictions of the global wave model, suggested by Nunez and Srinivasan (2014). One of the central assumptions is that the modulation density of action potentials is a function of cortical background excitability and inhibitory feedback strength. Quantification of the model predicts that an increase in parameter β (reflecting the degree of cortical background excitability) is associated with an increase in oscillatory amplitude but a decrease in frequency. This is exactly the pattern of results, we found for the peri- and poststimulus interval at the leading electrode. Our findings are to our knowledge the first for the human scalp EEG, showing cycle per cycle fluctuations of amplitude and period in alpha, resembling closely findings on rat hippocampal gamma (Whittington et al., 1995; Traub et al., 1996; Atallah and Scanziani, 2009).

For the physiological interpretation of traveling waves, different conceptualisations have been used. As an example, spatial phase shifts may originate from a phase lag between neighboring, coupled neuronal oscillators (Ermentrout and Kleinfeld, 2001; Wu et al., 2008). Here, however, we refer to the water wave analogy, Nunez (2000) uses to illustrate the dynamics of cortical traveling waves, spreading via myelinated fibers that connect neighboring cortical regions. This model has the advantage that it has a strong emphasis on large scale white matter connectivity, which presumably plays also an important role for cognitive processes and the scalp EEG as well (Hipp et al., 2012). It should be noted, however, that this model, which assumes fast intercortical propagation has been challenged recently. Hindriks et al. (2014) point out that local field potential recordings in animal studies show slow propagation velocities that suggest intra- instead of intercortical propagation. Using a combination of EEG analysis and biophysical modeling, the authors demonstrate that the fast scalp velocities can be accounted for by slow traveling oscillations. The central idea is that a slow traveling movement in a small cortical region may ‘project’ via volume conduction to a large scalp region, thereby mimicking a fast traveling movement. For our results, this would mean that the evoked traveling alpha movement would be restricted to a comparably small cortical region.

With respect to the functional meaning of the evoked traveling alpha wave, we have suggested that it may reflect a top down controlled process that is associated with an early categorization of the presented stimulus (Klimesch, 2011). Because for words, topographical P1 latencies are sensitive to lexical and (possibly already) semantic features (Zauner et al., 2014), it was suggested that the evoked traveling alpha wave reflects early stages of access to lexical and possibly also semantic memory. The posterior topography may be associated with the processing of visual and graphemic features that enable access to lexical and semantic memory.

Our findings suggest a close association between alpha and the P1. This, however, does not mean that other frequencies do not play an important role. Higher frequencies, particularly in the beta (e.g., Gruber et al., 2005) and gamma range (e.g., Porcaro et al., 2011) may also transiently phase align with alpha within the P1 latency window.

## Conclusion

The important conclusion is that single trial fluctuations in amplitude size are associated with fluctuations in the length of alpha periods in the peri- and prestimulus period. These fluctuations modulate alpha frequency and are capable of inducing a traveling trajectory that may be interpreted as a spreading activation process within a neural network that is associated with access to memory. We assume that the topography of prestimulus amplitude increases reflects a top down process that controls the poststimulus flow of spreading activation, as manifested by evoked traveling alpha waves.

## Author Contributions

WK and AZ designed the study; CB and NH performed all recordings; CB performed the ERP analysis; NH performed the single trial analyses; AZ, WG, and JL helped in analyzing EEG data; WK and HK contributed in interpretation of EEG data; and WK, CB, and NH wrote the manuscript. All authors approved the final manuscript.

## Conflict of Interest Statement

The authors declare that the research was conducted in the absence of any commercial or financial relationships that could be construed as a potential conflict of interest.

## Abbreviations

NOF+: high number of features
NOF-: low number of features
